# Hydroxyapatite Formation on a Novel Dental Cement in Human Saliva

**DOI:** 10.5402/2012/624056

**Published:** 2012-09-27

**Authors:** Johanna Engstrand, Erik Unosson, Håkan Engqvist

**Affiliations:** Division of Applied Materials Science, Department of Engineering Sciences, The Ångström Laboratory, Uppsala University, P.O. Box 534, 751 21 Uppsala, Sweden

## Abstract

Dental materials have to meet high standards regarding mechanical strength and handling properties. There is however only a limited amount of research that has been devoted to natural formation of hydroxyapatite (HA) in contact with the materials. The objective of the current investigation was to study the surface reactions occurring in human salvia on a novel dental cement. Ceramir Crown & Bridge, a bioceramic luting agent intended for permanent cementation of conventional oral prosthetics, was evaluated by immersing discs made from the cement in human saliva and phosphate buffered saline (PBS) for seven days, after which they were dried and analyzed. The analytical methods used in order to verify HA formation on the surface were grazing incidence X-ray diffraction (GI-XRD), scanning electron microscopy (SEM), and X-ray photoelectron spectroscopy (XPS). All results showed that HA was formed on the surfaces of samples stored in saliva as well as on samples stored in PBS. The possibility of a dental luting cement to promote natural formation of HA at the tooth interface increases the stability and durability of the system and could help prevent secondary caries.

## 1. Introduction

Publications describing dental materials that promote restoring of a damaged tooth based on natural formation of hydroxyapatite (HA) are limited, that is, functional remineralizing/bioactive dental materials. All clinically used dental materials have to meet high standards with respect to handling and mechanical properties. However, the known bioactive biomaterials generally have low mechanical strength or are difficult to handle and mould, for example, bioglasses [1–3]. Attempts have been made to develop materials that promote dental remineralization of the tooth by releasing Ca^2+^ and PO_4_^2−^ ions, but so far no truly bioactive dental material has been made commercially available [4]. One material that is considered to have remineralizing properties and the necessary mechanical properties to function as a dental material is calcium aluminate (CA) [5, 6]. The main mechanism behind bioactive properties of a material has been connected to its negatively charged surface and its release of calcium, phosphate, and hydroxyl groups. These properties can even promote HA formation on the surface of the material when stored in water [7]. The mechanisms behind the HA surface layer formed have been discussed in detail elsewhere [8–10]. There are a number of materials that display bioactive properties, normally aimed as bone void fillers, for example, bioglasses, sintered hydroxyl apatites, wollastonite, and calcium phosphates. As mentioned above, it has recently been demonstrated that calcium aluminates (CAs) also have bioactive/remineralizing properties in simulated body fluids (SBFs) and *in vivo* [11–13]. Normally, testing of the ability of a material to promote HA formation is performed in SBF [14]. In this paper, the possible HA formation on the CA-based dental cement Ceramir Crown & Bridge was studied in human saliva and was compared to storage in phosphate buffered saline (PBS, similar to SBF, but with higher amounts of phosphates present). There are a number of unknown factors that such testing can clarify, for example, if the ionic concentration in human saliva is enough for HA formation, and possible crystallinity as well as morphology of the formed HA.

One current hypothesis is that the use of remineralizing materials in dentistry would prevent secondary caries due to a natural formation of apatite between material and tooth, leading to a stable interface.

## 2. Materials and Methods

### 2.1. Test Materials

The material under evaluation in this study was Ceramir Crown & Bridge (Ceramir C&B, Doxa Dental AB, Uppsala, Sweden; composition in Table 1). Ceramir C&B is a bioceramic luting agent intended for permanent cementation of conventional oral prosthetics. The materials performance as a dental luting agent has been evaluated elsewhere [15–22] and is not part of the scope. The material is a combination of a hydraulic cement system (calcium aluminate, CA) and a glass ionomer cement. Ceramir C&B stored in PBS was used as a positive control [23], and poly(methyl methacrylate) (PMMA) stored in saliva was used as a negative control.

### 2.2. Storage Solutions

Dulbecco's D 8662 phosphate buffered saline (PBS) was used as received from Sigma-Aldrich (St. Louis, MO, USA). The composition is shown in Table 2. Whole saliva was collected from two females and one male between 24 and 27 years old. The gathering of saliva took place at 10.30 am, at which point the subjects had not been eating or drinking for at least two hours. As stimulus for saliva production, the subjects chewed on Parafilm “M” laboratory film (Pechiney Plastic Packaging, Chicago, IL, USA). The saliva collected during the first minute was discarded. In total, 75 mL was collected during 25 minutes. 

### 2.3. Sample Preparation and Storage

Ceramir C&B was mixed according to the instructions given by the manufacturer. Cylindrical molds with diameter 8 mm and height 3 mm were filled with cement paste using a stainless steel spatula. The cement was then allowed to set for 10 minutes at 37°C and >90% humidity, after which the samples were removed from the molds and gently polished on one side using 1200 grit SiC paper. Samples were subsequently transferred to water-containing beakers where they were kept for three hours at 37°C for complete setting. Since the amount of saliva available was limited, six samples were transferred to individual 20 mL Falcon-tubes containing 3 mL of freshly collected saliva (samples hereafter known as CBS). An additional six samples were individually put in 20 mL Falcon-tubes containing 18 mL PBS (samples hereafter known as CBP). Falcon-tubes, with their conical shape, were chosen as storage containers in order to expose all sides of the samples to the storage solutions. According to ISO standard 23317 [14] for testing bioactivity, samples should be stored in their untouched storage solution for the desired amount of time. Although saliva can degrade during storage, the authors chose not to change the saliva continually during the test to resemble the standard procedure. Therefore, the samples were kept in their untouched solutions for seven days in an oven (Carbolite, Hope Valley, UK) at 37°C after which they were gently washed with distilled water and dried at room temperature. A bulk sample was obtained by grinding down a CBP sample using 640 and 1200 grit SiC paper, leaving no trace of the previous surface layer. The reference PMMA samples were treated the same way as the CBS samples. These were cut from a rod with a diameter of 8 mm into 3 mm thin samples, the same size as the mold used for casting the Ceramir C&B samples.

### 2.4. Saliva and PBS Analysis

The concentration of phosphates was measured for both storage solutions by flow injection analysis UV-VIS (ALS Scandinavia AB, Täby, Sweden) according to the CSN ISO 15681-1 standard. The detection limit of the equipment was 0.04 mg/L. The pH of saliva and PBS was approximated using Hydrion Brilliant pH dip stiks (Sigma-Aldrich, St. Louis, MO, USA) with a measuring range from pH 1–14.

### 2.5. Scanning Electron Microscopy (SEM)

The morphology of the samples was investigated using a scanning electron microscope (LEO 1550, Carl Zeiss, Oberkochen, Germany). Prior to the SEM analysis a thin gold/palladium coating was sputtered onto the surface.

### 2.6. X-Ray Diffraction (XRD)

A Siemens D5000 diffractometer (Bruker AXS Gmbh, Karlsruhe, Germany) was used for grazing incidence X-ray diffraction (GI-XRD) measurements of the surface crystal structure. The analysis was set up with a beam incidence angle of 2° and a 2*θ* scan between 25 and 33 degrees, where the most intense peaks for HA (Ca_10_(PO_4_)_6_(OH)_2_) and monocalcium aluminate (CaAl_2_O_4_) are found.

### 2.7. X-Ray Photoelectron Spectroscopy (XPS)

The surface composition was analyzed by X-ray photoelectron spectroscopy (Al K*α* X-ray source, Quantum 2000, Physical Electronics, Inc., Chanhassen, MN, USA,). XPS survey spectra were acquired and recorded using 100 eV. For quantitative analysis the Ca2p, P2s, O1s, Al2p, Si2p, Sr3p, and F1s peaks were used. Peaks from Sr3d and P2p electrons were not used as these overlap at 135 eV, making distinction between them virtually impossible. Analysis was performed on two random sites on CBS, and five sites each on CBP and bulk surface after being sputtered for 5 s with 2 kV.

## 3. Results

### 3.1. Analysis of Saliva and PBS

Although literature states that CO_2_ loss from saliva can increase pH after some days [24] only minor differences were observed when comparing the pH of saliva and PBS before and after storage, see Table 3. This is likely due to the fact that the saliva was stored in closed containers with walls and lid impermeable for CO_2_, preventing CO_2_ losses [24]. Soaking the samples resulted in a significant phosphate decrease of roughly 300 mg/L in both buffer and saliva. Since no big pH change was observed, the precipitation was not likely due to an increase in pH. Accounting for the differences in solution volume, this corresponded to an HA deposition of approximately 1.5 mg on each CBS disk and 7.9 mg on each CBP disk. A minor decrease in phosphate content was noted also for the PMMA sample.

### 3.2. SEM

**Figure 1:**
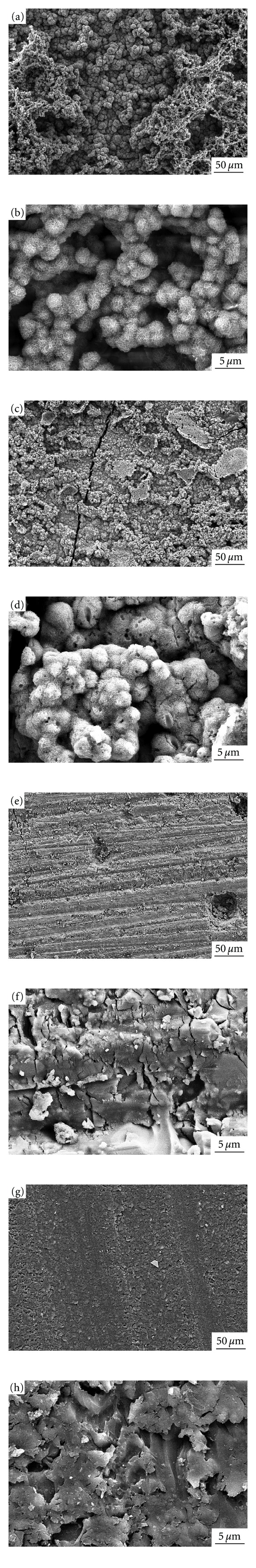
(a) and (b) CBP, (c) and (d) CBS, (e) and (f) PMMA reference, and (g) and (h) bulk material. In (c) cracks are visible (most likely due to drying during vacuum pumping).

Representative sample images are shown in Figure 1. Both CBS and CBP showed agglomerates of spherical particles. Both large and small particles were present on the CBP surface, where the smaller were close in size with particles found on the CBS surface. Crystal morphology on the other hand was different; crystals on CBP showed a plate-like structure while crystals on CBS were more needle-like. Channels were visible in the particles on CBS, which were most likely due to enzymes and other components of the saliva that are not present in PBS. Since no bulk material is observable on CBP, but some is visible on CBS, the deposited layer on CBP is deemed thicker than the one on CBS. The bulk reference sample only bore marks of grinding. PMMA plates showed no signs of film growth.

### 3.3. XRD

**Figure 2:**
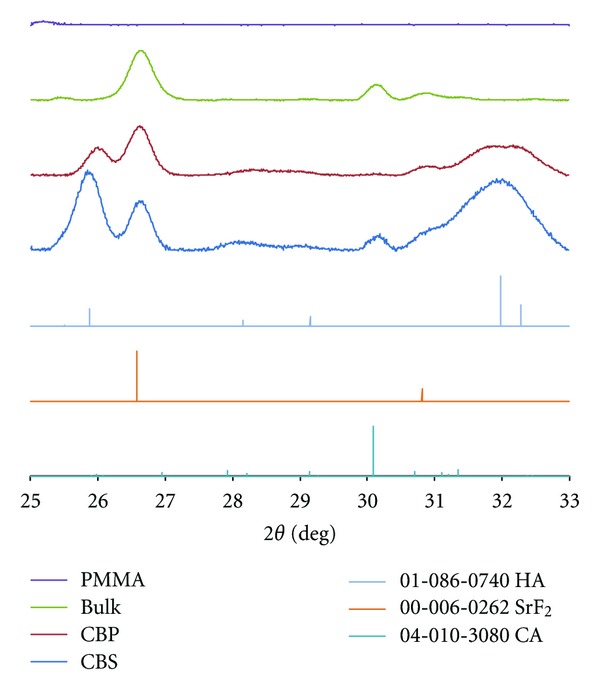
GI-XRD data for analyzed materials. Spectrums from CBS and CBP samples, the bulk reference and the PMMA reference are included in the figure. Patterns for CA (PDF#01-070-0134), HA (PDF#01-086-0740), and SrF_2_ (PDF#00-006-0262) are included. Broad peaks of low intensity between 28 and 29 degrees are from Gibbsite (PDF#01-074-1774) and Katoite (PDF#00-024-0217).

All cement samples exhibited peaks corresponding to SrF_2_, a minor constituent of the cement showing high crystallinity, see Figure 2. Clear presence of HA was only detected on CBS and CBP samples, where also traces of the hydrates gibbsite and katoite were found. A major peak of CaAl_2_O_4_ was visible in the bulk sample and CBS sample, while it was not as prominent in the CBP sample. This indicated that the CBP deposited layer was thicker than the layer on CBS. Only background noise was detected when analyzing the PMMA reference sample stored in saliva.

### 3.4. XPS

Results from the XPS analysis showed that the surface layer compositions of both CBS and CBP differ from bulk composition (Table 4). The ratios between calcium and phosphorous were 1.55 ± 0.06 and 1.82 ± 0.23 for the surface layers on CBS and CBP, respectively, clearly indicating presence of HA. Analysis also showed presence of aluminum on the CBP surface, whereas CBS did not.

## 4. Discussion

When describing the chain of events during hardening of Ceramir C&B luting cement at the material-tooth interface, the most important information is found in the hardening chemistry of CA (calcium aluminate). CA is a hydraulic cement widely used in industrial applications and is well investigated [25–30]. The hardening is a series of chemical reactions taking place during a dissolution-precipitation process. The CA in Ceramir C&B is in principle monophasic CaAl_2_O_4_, and the chemical reaction with water is shown in (1), a reaction where pH increases and can reach as high as 11. The two principal end products of this reaction are the hydrates katoite and gibbsite:

(1) 3 CaAl2O4+12 H2O  →  Ca3(Al(OH)4)2(OH)4  (katoite)        +4 Al(OH)3  (gibbsite).

After setting of the cement, precipitation of other species on the cement surface is controlled by solubility products of respective species, pH of the environment, and the concentration of other ions that are present. For instance when placed in contact with body fluids, phosphate and carbonate ions are readily available and calcium carbonates and calcium phosphate may precipitate in the contact zone of CA, a mechanism described in [12]. The ions involved may come either from the material itself or from the surrounding environment.

When used as a luting agent, hydrophilicity and viscosity of the cement will determine how well it wets the tooth surface. During hardening, the initial ceramic powder will dissolve and the resulting ion solution will penetrate irregularities of the tooth wall. When precipitation occurs it does so mainly in the form of nano-sized hydrates on pre-existing nuclei and filler particles on the tooth wall. During this process, the irregularities will be filled predominantly by CA hydrates. However, other species will also be formed at the interface, depending on the concentration of ions present. This means that the mineral density at the material-tooth interface will increase [12], resulting in a mineralization. For a distinct layer of HA to be precipitated between the material and the tooth, phosphate ions must be present on the tooth surface. 

The combination of CA with a glass ionomer, as in Ceramir C&B, gives a cement that is initially dominated by the glass ionomer setting reaction, having a slightly acidic pH. The initial pH is around 5 and reaches neutrality after approximately 30 min at 37°C. After roughly 12 h at the same temperature, it ends up at about 8.5. Adding a glass ionomer also changes the rate and composition of the initial ion leakage from the material. The acid soluble glass, being one of the main constituents of a glass ionomer, contains both phosphorus and strontium oxide, which gives a leakage of Sr^2+^ and PO^4-^ during hardening. These additional ions change the path of precipitation and may give alterations to precipitated species, as well as to their morphology. For instance strontium ions are known to create strontium substituted HA that has a different morphology than “normal” HA [31]. When the material is fully hardened and the interface has been created, remodeling is limited. However, the liquid present in the dentinal tubuli could cause remodeling of the material inside each tubuli. Depending on local conditions, that is, pH and ion concentration, the species precipitated will vary. Short-term retention of Ceramir C&B has been proven in [22]. Moreover, a clinical study performed by Jefferies et al. showed no retention problems after three years; the results from the one- and two-year recalls are presented in [16] and [32], respectively. The low solubility of HA (pKs = 58) together with the negatively charged surface of the cement creates an excellent environment for HA growth on the surface. It has previously been shown [23] that the components of the cement investigated together with PBS are sufficient for formation of a HA layer on the cement surface.

In this study stimulated whole saliva was used since it can be readily collected and well resembles an *in vivo* situation where contributions from all salivary glands are accounted for. Stimulated saliva contributes to as much as 90% of average daily salivary production [33]. Whole saliva refers to the fluid normally present in the mouth and consists of a complex mixture of contents derived from major and minor salivary glands, as well as various nonsalivary components such as gingival crevicular fluid, bacteria, and food debris [34, 35]. Measurements of the phosphorous content of both the stimulated whole saliva and the PBS showed that the stimulated whole saliva contains enough phosphates to promote a good environment for HA growth, similar to the mechanism that has been described for PBS [23].

The XRD data presented in this study showed that the main crystalline species of bulk material are SrF_2_ and CaAl_2_O_4_, while the remaining peaks can be assigned to species located on the surface of the material. The clear HA signals from both CBS and CBP samples proved the HA formation on cement surface in both saliva and PBS. Most of the phosphate ions in the saliva have been deposited on the ceramic surfaces, as shown in the analysis of the composition of the storage solutions. From SEM micrographs (Figure 1) the deposited layer on CBS is deemed thinner than that on CBP. If the same amount of saliva as PBS had been used, there might have been a thicker HA layer or another composition on the CBS surface.

The increased amount of calcium and phosphate compared to bulk and the ratios between them (Table 4) indicate that a HA layer was formed on the surface in both groups. For the CBP group, the high aluminum content together with increased calcium to phosphate ratio suggests formation of some calcium aluminate compounds (katoite and gibbsite) on the surface.

As discussed above, a certain set of prerequisites needs to be fulfilled in order to get HA precipitation on a material surface; the correct ions must be present in high enough concentrations, the pH must be in the correct range, that is, alkaline, and a negatively charged surface is necessary [8–10]. In reviewing commercially available dental cements and their chemistries, Ceramir C&B is the only one fulfilling the above-mentioned necessary prerequisites. Zinc phosphates are too acidic and do not contribute with Ca^2+^. Resin-based materials are acidic or neutral, but so far not alkaline, and they do not show extended ion leakage. Glass ionomers have an ion leakage but are acidic and cannot induce HA formation on its surface. To date, the only other material systems used in dentistry that would have this feature is the *mineral-trioxide-aggregate*- (MTA-) based materials [36, 37] and possibly the calcium-hydroxide-based materials.

The results from this study showed that the CA-based dental cement Ceramir C&B provided a good environment for HA growth. Furthermore, human saliva was shown to contain sufficient amounts of calcium and phosphate ions to induce HA growth on the cement surface. 

## Figures and Tables

**Table 1:** Composition of Ceramir C&B powder.

Component	Amount (wt%)
Tartaric acid	1–3
Strontium fluoride	4–6
Poly(acrylic acid)	10–15
Acid soluble glass	32–37
(SiO_2_-Al_2_O_3_-SrO-P_2_O_5_-NaO_2_-F^−^)	
Ground calcium aluminate	45–55

**Table 2:** Composition of the PBS used, Dulbecco's D 8662 phosphate buffered saline.

Salt	Amount (g/L)
CaCl_2_·2H_2_O	0.133
MgCl_2_·6H_2_O	0.1
KCl	0.2
KH_2_PO_4_ (anhydrous)	0.2
NaCl	8
Na_2_HPO_4_ (anhydrous)	1.15

**Table 3:** pH and phosphate content of saliva and PBS, before and after storage.

Sample	pH	Phosphate content (mg/L)
Saliva before soaking	7.5	330
Saliva after 7 days with Ceramir C&B	8	22.3
Saliva after 7 days with PMMA plates	8	263
PBS before soaking	7	909
PBS after 7 days with Ceramir C&B	7	595

**Table 4:** Atomic percent of elements obtained by XPS analysis of CBS, CBP, and bulk reference. Number of measured points for each sample is indicated inside parenthesis. <DL indicate amounts less than the detection limit of the instrument.

	O1s	Ca2p	P2s	Sr3d	Al2p	F1s	Si2p
CBS (2)	55.8 ± 0.9	26.0 ± 0.2	16.8 ± 0.7	1.5 ± 0.1	<DL	<DL	<DL
CBP (5)	55.7 ± 2.6	20.6 ± 2.1	11.4 ± 1.6	1.3 ± 0.3	11.0 ± 4.6	<DL	<DL
Bulk (5)	50.8 ± 1.8	9.5 ± 0.5	0.0 ± 0.1	2.3 ± 0.3	30.1 ± 1.8	3.8 ± 0.7	3.5 ± 0.2
